# The Manchester Meeting

**Published:** 1911-07-08

**Authors:** 


### HOSPITALS AND STATE INSURANCE.

### THE MANCHESTER MEETING.

### ADOPTING THE ASSOCIATION'S MEMORIAL.

A meeting of representatives of the voluntary hospitals of Lancashire and Cheshire was held at the Manchester Town Hall on Tuesday, June 27 last, to consider the National Insurance Bill and to receive the memorial which is being presented to the Chancellor of the Exchequer on behalf of the voluntary hospitals of Great Britain and Ireland by the British Hospitals' Association.

Sir William Cobbett, of the Manchester Royal Infirmary, occupied the chair, and the following delegates were present: Mr. F. W. Peel, Mr. H. H. Smith-Carrington, Mr. Wm. Stones, Mr. Russell Allen, Mr. Jno. Cooper, Col. W. W. Clapham, and Mr. W. G. Carnt, Manchester Royal Infirmary; Mr. P. W. Kessler and Mr. W. S. Prophet, Manchester Royal Eye Hospital; Dr. T. H. Pinder, Manchester Ear Hospital; Mr. Wm. Buckley and Mr. H. J. Eason, Manchester Children's Hospital; Mr. Jas. Ferguson, Northern Counties Hospital for Incurables, Mauldeth, Manchester; Mr. R. G. Lawson, Mr. Fredk. P. Nathan, and Mr. N. W. Smith-Carington, Christie (Cancer) Hospital, Manchester; Sir F. Forbes Adam and Mr. R. Ratcliffe, St. Mary's Hospital, Manchester; Mr. Herbert Teague, Manchester Northern Hospital; Mr. C. E. Marshall, Manchester Dental Hospital; Mr. W. Armitage and Mr. John R. Oliver, Ancoats Hospital, Manchester; Mr. J. T. Loewy, Victoria Memorial Jewish Hospital, Manchester; Mr. Jno. T. Lingard, St. John's Hospital for Ear and Eye, Manchester; Sir D. Gamble, Bart., St. Helen's Hospital; Mr. Wm. P. Park, J.P., Preston Royal Infirmary; Mr. L. Pilkington, Mr. Fredk. Burton, and Mr. G. Ruddle, Salford Royal Hospital; Mr. Sewart (represented by Mr. Carnt), Royal Lancaster Infirmary; Mr. Wm. Barlow and Mr. W. Stockdale, Aitken Hospital, Ramsbottom; Mr. C. Barnes and Mr. W. Thraves, Chesterfield and North Derbyshire Hospital; Major C. Tyler and Mr. Tom Kay, Stockport Infirmary; Mr. Allen Naldrett, Royal Southern Hospital, Liverpool; Mr. W. W. Cannon, Mrs. Knowles, and Mrs. Harwood, Bolton Infirmary; Mr. N. A. Smith, Blackburn Infirmary; Capt. Crossfield, Mr. W. Peter Rylands, Mr. Alderman Burton, and Mr. J. H. Smethurst, Warrington Infirmary; Dr. J. R. Gaylord, Birkenhead Maternity Hospital; Mr. J. H. Shaw and Rev. E. C. Collier, Southport Infirmary; Mr. H. Wade Deacon, David Lewis Northern Hospital, Liverpool; Mr. Jno. Hossall, Rev. Canon Binney, and Mr. J. A. Cowley, Northwich Infirmary; Mr. Alderman Shaw, Mr. Maurice Lees, and Mr. F. Oliver, Ashton-under-Lyne Infirmary; Dr. Golland and Dr. H. F. Ransome, Altrincham Hospital; Mr. J. W. Shaw and Mr. Reginald J. Spencer, Bury Infirmary; Mr. F. Taylor, Urmston Cottage Hospital; Mr. A. Langham, Victoria Hospital, Accrington; Mr. W. Stevenson, Devonshire Hospital, Buxton; Mr. Alderman Saml. Turner and Mr. H. Booth, Rochdale Infirmary; Mr. H. E. Hanrahan, Macclesfield Infirmary; Mr. Adam G. Rankine and Mr. Alderman Shelmerdine, Liverpool Maternity Hospital; Mr. Holford Harrison, Royal Infirmary, Liverpool; Mr. J. C. Styles, North Lonsdale Hospital, Barrow-in-Furness; Mr. P. Loftos, Victoria Hospital, Blackpool. The Victoria Hospital, Burnley, was also represented.

There were seventy-two representatives present, representing the following twenty-six towns: Manchester and Salford, Liverpool, Warrington, Lancaster, Preston, Blackburn, Ashton-under-Lyne, Buxton, Chesterfield, Stockport, St. Helens, Burnley, Rochdale, Birkenhead, Bolton, Barrow-in-Furness, Southport, Macclesfield, Bury, Accrington, Northwich, Altrincham, Blackpool, Urmston, Ramsbottom.

### Sir William Cobbett's Speech.

According to the *Manchester Courier*, from which we take the greater part of the following report, Sir Wm. Cobbett, in opening the meeting, said they were called together in consequence of a request made at a meeting of hospital representatives held in London under the auspices of the British Hospitals Association. The question in which they were all interested was the effect which the National Insurance Bill would have on the funds of the voluntary hospitals and what remedy the hospitals would suggest to the Minister in charge of the Bill as being likely to remove the difficulties which would arise if the Bill was passed into law. The meeting in London was attended by upwards of two hundred hospital representatives, and the result of the discussion was that a sub-committee was appointed with power to act. That committee had met on two occasions—on June 16 and on Monday last—and as the result of their deliberations a memorial was adopted, which it had been decided should be presented to the Chancellor of the Exchequer by a deputation at the earliest possible moment. The Chancellor of the Exchequer must have contemplated that the hospitals would continue to extend to the suffering poor those benefits which they have hitherto done, and the conclusion he came to was that hospitals would not be affected. But, unfortunately, there was arrayed against him the unanimous opinion of all hospital governors and managers that they would be affected.

### Voluntary Hospitals Under the Bill.

That was the opinion of a very large body of experienced men who had been engaged for years in devoting their time and energies for the advancement of hospitals, and surely it was a piece of weighty testimony which ought to have its effect not only with the Chancellor of the Exchequer but with Parliament. The contributions of workmen and employers formed a large proportion of voluntary contributions to all hospitals, and in most cases the workmen's contributions were most important. The Chancellor was taking fourpence from the workman and threepence from the employer, and the effect of this would be to divert at the source the contributions which the workmen and the employers previously made. A workman who had been accustomed to subscribe to the Hospital Saturday Fund would quite naturally say, "I think I have paid enough. I cannot afford to pay more." The employer would have the same argument. He (the Chairman) could not understand, under those circumstances, why the Chancellor of the Exchequer should say, as he had said more than once, that the hospitals were not affected. He thought that if anyone were to put forward a scheme which involved the diversion of the Thirlmere water into the Irish Sea the citizens of Manchester would say that the water supply was affected. Our hospitals had been compared with similar institutions in Germany. He was told that the hospital system in Germany differed considerably from that in England. In Germany both rich and poor went to the hospitals for treatment, and they paid according to their means. The sum total of voluntary contributions was not very large, and they came in the form of gifts for special purposes. It was therefore quite impossible to compare a German hospital with any English voluntary hospital.

The treatment in a hospital of disease and accident of insured persons should be made a benefit and placed upon the same footing in the Bill as tuberculosis treated in sanatoria, and clause 8 (1 *b*) should be amended accordingly; also clause 15 (1) should be amended so as to direct health authorities to make similar arrangements with persons having the management of a hospital for the treatment therein of insured persons entitled to hospital benefit, as the clause now directs health committees to make with persons having the management of sanatoria for the treatment of insured persons entitled to sanatorium benefit. They did not ask for State aid, for the moment they began to talk about State aid the death-knell of voluntary hospitals was sounded. What the hospitals do ask for is that they may have their share of the workman's fourpence and the employer's threepence. A State insurance scheme, in order to be beneficial, must not cripple the hospitals.

The memorial was then submitted to the meeting. It contains the following provisions—(1) Any adequate scheme for ensuring medical attendance for the working classes of the kingdom involves the provision of hospital accommodation for persons suffering from serious or unusual diseases or standing in need of operative assistance. (2) The voluntary hospitals of the kingdom are at present supplying this need at an annual cost of nearly four million pounds, exclusive of interest on the original cost of the hospital buildings. (3) The greater part of these funds is obtained from legacies, donations, or annual subscriptions, to which all classes of the community contribute. (4) All the medical schools of the country are worked in connection with and depend for their efficiency on voluntary hospitals, and such medical schools have supplied the only opportunities for training to the physicians and surgeons of the country. Such training has been extremely efficient, and those trained thereby compare favourably with the medical men of any other nation. (5) The voluntary hospitals have hitherto been the chief centres of research and of widening the possibilities of medical and surgical treatment. Many hospitals have already great difficulty in raising funds to meet their annual expenditure, and any reduction of the means at the disposal of the managers of voluntary hospitals will necessitate a corresponding limitation of the work which they can undertake. (6) The National Insurance Bill contains no provision which is calculated to increase the resources of voluntary hospitals, and it is apprehended that if such Bill is passed in its present form a large reduction in the contributions of both employers and employed will immediately ensue. (7) The persons insured by the Bill will in the future, as they do now, come to voluntary hospitals for treatment in cases of serious and obscure illness and cases requiring surgical treatment. The memorialists therefore urge that the benefits to be conferred by the Bill should be extended to cover treatment in general or special hospitals, and that arrangements should be made to recoup all such hospitals for the expenses which they may actually incur in the treatment of persons voluntarily or compulsorily insured by the Bill.

Sir Frank Forbes Adam, in moving a resolution expressing approval of the memorial, said he thought the Chancellor of the Exchequer should deal very tenderly with a representation coming from such a source as that. The Chancellor had said that he wanted to do something to improve the condition of those who were downtrodden. The hospitals had for many years past been carrying on the work which the Chancellor wished to extend to other classes.

Alderman W. P. Park, Chairman of Preston Royal Infirmary, said this hospital would lose at least £2,000 if the Bill became law.

Mr. J. A. Cawley, Secretary of the Victoria Fund, Northwich, did not think the payments suggested in the memorial would be sufficient to meet the deficiency that would arise.

After further discussion the resolution was carried unanimously. A further resolution, moved by Mr. Cawley, urging that ample time should be given for the consideration of the Bill by the hospital authorities, was defeated.

